# A Systematic Review and Meta-Analysis of Male Infertility and the Subsequent Risk of Cancer

**DOI:** 10.3389/fonc.2021.696702

**Published:** 2021-10-14

**Authors:** Samira Behboudi-Gandevani, Razieh Bidhendi-Yarandi, Mohammad Hossein Panahi, Mojtaba Vaismoradi

**Affiliations:** ^1^ Faculty of Nursing and Health Sciences, Nord University, Bodø, Norway; ^2^ Department of Biostatistics, University of Social Welfare and Rehabilitation Sciences, Tehran, Iran; ^3^ Department of Epidemiology, School of Public Health and Safety, Shahid Beheshti University of Medical Sciences, Tehran, Iran

**Keywords:** melanoma, prostate cancer, risk, testicular cancer, male infertility

## Abstract

**Objectives:**

The primary objective of this systemic review and meta-analysis was to investigate the risk of developing composite outcome of all cancers, regardless of the type of cancer among men with infertility diagnosis compared to fertile counterparts. The secondary objective was to compare the pooled risk of developing individual specific cancers between two groups.

**Methods:**

A systematic literature search was performed on the databases of PubMed (including Medline), Scopus, and Web of Science to retrieve observational studies published in English language from 01.01.1990 to 28. 02. 2021. They assessed cancer events in males with an infertility diagnosis compared to controls without infertility. The outcomes of interest were a composite outcome of cancers including all known cancer types, and also specific individual cancers. The fixed/random effects model was used to analyze heterogeneous and non-heterogeneous results. Publication bias was assessed using the Harbord test, Egger test, Begg test, and funnel plot. The pooled odds ratio of cancers was calculated using the DerSimonian and Laird, and inverse variance methods. Studies’ quality and risk of bias were assessed using structured standard tools.

**Results:**

We included eight cohort studies involving 168,327 men with the diagnosis of infertility and 2,252,806 men without it. The total number of composite outcome of cancers as well as individual cancers including prostate, testicular and melanoma were 1551, 324, 183 and 121 in the infertile men and 12164, 3875, 849, and 450 in the fertile men, respectively. The pooled OR of the composite outcome of cancers, regardless of the type of cancer, in men with infertility was 1.4 folds higher than those without infertility (pooled OR = 1.43, 95% confidence interval [CI]: 1.25-1.64). Meta-analysis of individual cancers including prostate, testicular and melanoma between two groups was carried out. The pooled ORs of testicular and prostate cancers in men with the diagnosis of infertility were significantly higher than controls without infertility (pooled OR = 1.91, 95% CI: 1.52-2.42 and pooled OR = 1.48, 95% CI: 1.05-2.08, respectively). Additionally, the pooled OR of melanoma in men with infertility was 1.3 folds higher than those without infertility (pooled OR = 1.31, 95% CI: 1.06-1.62).

**Conclusion:**

A greater risk of cancers in men with male infertility was found suggesting that the history of male infertility might be an important risk factor for developing cancers in later life. Further well-designed long-term population-based prospective studies, considering all known cancers and their accompanying risk factors should be conducted to support our findings.

## Introduction

Infertility is defined as failure to achieve a clinical pregnancy after 12 months or more of regular unprotected sexual intercourse (1). Male infertility is solely responsible for 10-30% of infertility cases and contribute to 50% of overall infertility cases (2, 3). Although male infertility is not a reportable disease, the manifestations of male infertility can signify a future health concern (4–6). The growing body of literature suggests that male infertility can be a potential marker of contemporary or future medical diseases including cardiovascular metabolic and autoimmune disorders as well as mortality. However, exact mechanisms behind these associations remain elusive (6–10).

It has been reported that both genetics and environmental factors can play an important role in developing cancer among males suffering from infertility (9, 11). It is believed that male infertility, *per se*, may play as a risk factor for the development of genitourinary cancers in men (12). However, studies on cancers among men with infertility have reported controversial results. Mao et al., in a systematic review and meta-analysis of 11 studies reported that being childless was associated with a lower risk of prostate cancer (13). In contrast, in a recently published meta-analysis by Del Giudice et al. (12) on six population-based cohort studies, male infertility was associated with a subsequent risk of male-specific malignancies including testicular cancer and prostate cancer.

Given the lack of conclusive evidence regarding the risk of overall as well as individual cancers, the primary aim of our systemic review and meta-analysis was to investigate the risk of developing composite outcome of all cancers, regardless of the type of cancer among men with infertility compared to fertile men. The secondary objective was to compare the pooled risk of developing individual specific cancers between men with infertility compared to fertile men.

## Material and Methods

This systematic review and meta-analysis was performed by the Preferred Reporting Items for Systematic Reviews and Meta-Analyses (PRISMA) (14), to achieve the following objectives:

- to study the pooled risk of developing composite outcome of all cancers, regardless of the type of cancer among men with the diagnosis of infertility compared to fertile men;
- to compare the pooled risk of developing individual specific cancers between men with the diagnosis of infertility and fertile men.

The review question was framed using the PICO (population, intervention/Index, control, and outcomes) statement as follows: P: men with the diagnosis of male infertility; I: risk of developing cancer; C: men without male infertility; O: overall and individual cancer. Study protocol was developed before the study and was used as the guideline to conduct this research (**Supplementary Table 1**).

### Eligibility Criteria

All types of observational cohort studies including prospective, retrospective, and registered-based data studies assessing the risk of subsequent cancer development in men with the diagnosis of male infertility were eligible to be included in this systematic review and meta-analysis.

In addition, studies should have subjects without infertility as the control group; clearly define male infertility and cancers; report the number, prevalence, and risk of cancer in the groups.

The presence of preexisting cancer before male infertility diagnosis, and also the lack of any differentiation between male and female infertility led to exclusion. Also, gray literature and non-original studies including reviews, commentaries, editorials, letters, meeting abstracts, case reports, conference proceedings, governmental or organizational reports, dissertations, theses, unpublished data and presentations that did not provide accurate and clear data on research variables were excluded.

### Search Strategy

A comprehensive literature search was performed on the databases of PubMed (including Medline), Scopus, and Web of Science to retrieve observational studies published in English language from 01.01.1990 to 28.02.2021. They should have investigated the risk of the development of cancer in males with the diagnosis of infertility. Further, a manual search in the references list of selected studies and other relevant reviews was carried out to maximize the identification of eligible studies. The following keywords, alone or in combination, were used during the search process: (male infertility OR male sterility OR male sub-fertility OR azoospermia OR oligospermia OR semen quality OR fertility impairment) AND (cancer OR neoplasms OR neoplasia OR tumors OR carcino* OR onco* OR benign OR hyperplasia OR malignancies OR malignancy OR carcinoma) (**Supplementary Table 2**).

### Study Selection and Data Extraction

The titles, abstracts, and full texts of selected studies were screened independently by two review authors based on the eligibility criteria and the following data was extracted from eligible studies: first author’s name; journal title; publication year; country; study design; sample size; population characteristics including age and body mass index (BMI); definition of infertility; follow-up period; quality assessment; outcome measurements in terms of the number and prevalence of cancer. Any disagreement in the selection of studies was resolved through holding discussions between the authors and also seeking comments from the third review author. The data extraction process was double-checked to ensure the accuracy of data collection before the meta-analysis and prevent bias in data extraction and data entry.

### Outcome Measures

Primary outcome of interest was the pooled risk of developing composite outcome of all cancers, regardless of the type of cancer among men with the diagnosis of infertility. Secondary outcome was the pooled risk of developing individual specific cancers among men with the diagnosis of infertility. Male infertility was defined as men reporting the experience of infertility with the duration of more than one year (15).

### Quality Appraisal

Quality of the included studies was critically appraised in terms of the methodological structure and presentation of results. Two authors were made blind to the study’s author, country, and the journal’s title to evaluate the quality of each study independently. The quality of observational studies was assessed using the modification of the Newcastle–Ottawa Quality Assessment Scale for Non-Randomized Studies (NRS) (16). This scale contains 8 items within 3 main domains of selection, comparability and outcomes with the maximum and minimum scores of 9 and zero, respectively. Studies with scores above 6 were considered high quality, 4–6 moderate quality, and less than 4 low quality.

The Risk Of Bias in Non-randomized Studies (ROBINS) tool in non-randomized studies of interventions and observational studies was used to assess risk of bias (17) as the Cochrane Collaboration has recommended (18). Seven domains of (i) selection of exposed and non-exposed cohort, (ii) assessment of exposure, (iii) presence of the outcome of interest at the beginning of the study, (iv) control of prognostic variables, (v) assessment of the presence or absence of prognostic factors, (vi) assessment of outcome, and (vii) adequacy of follow-up were used for appraisal. The review authors judged the quality of each study and classified it into serious, critical, moderate, and low risk of bias and no information.

### Statistical Analysis

Meta-analysis was performed to evaluate the pooled OR (95% CI) of the outcomes of interest including individual cancers and composite of all cancers. Heterogeneity was evaluated using the I-squared (I^2^) statistics and values above 50% were interpreted as heterogeneity. Given the heterogeneous results of the included studies, the pooled effect was calculated using the random effect model. Publication bias was assessed through the visual inspection of funnel plot, Harbord test, Egger test, and Begg test. When the funnel plot is symmetrical and the p values of Harbor test, Begg test, and Egger test are >0.05, no significant publication bias exists in the meta-analysis. Pooled OR (95% CI) was estimated using the DerSimonian and Laird, and inverse variance methods. Forest plots were drawn to show the estimation of pooled OR (95% CI) in the included studies. Sensitivity analysis was run to investigate the influence of each individual study on the estimation of overall meta-analysis summary. The graph of the results of an influence analysis in which the meta-analysis was re-estimated, omitting each study in turn, was drawn. Significant level was considered p <0.05 and all statistical analyses were performed using the STATA software (version 14; STATA, INC., College Station, TX, USA).

## Results

### Search Results, Study Selection, Study Characteristics, and Quality Assessment

**Figure 1:**
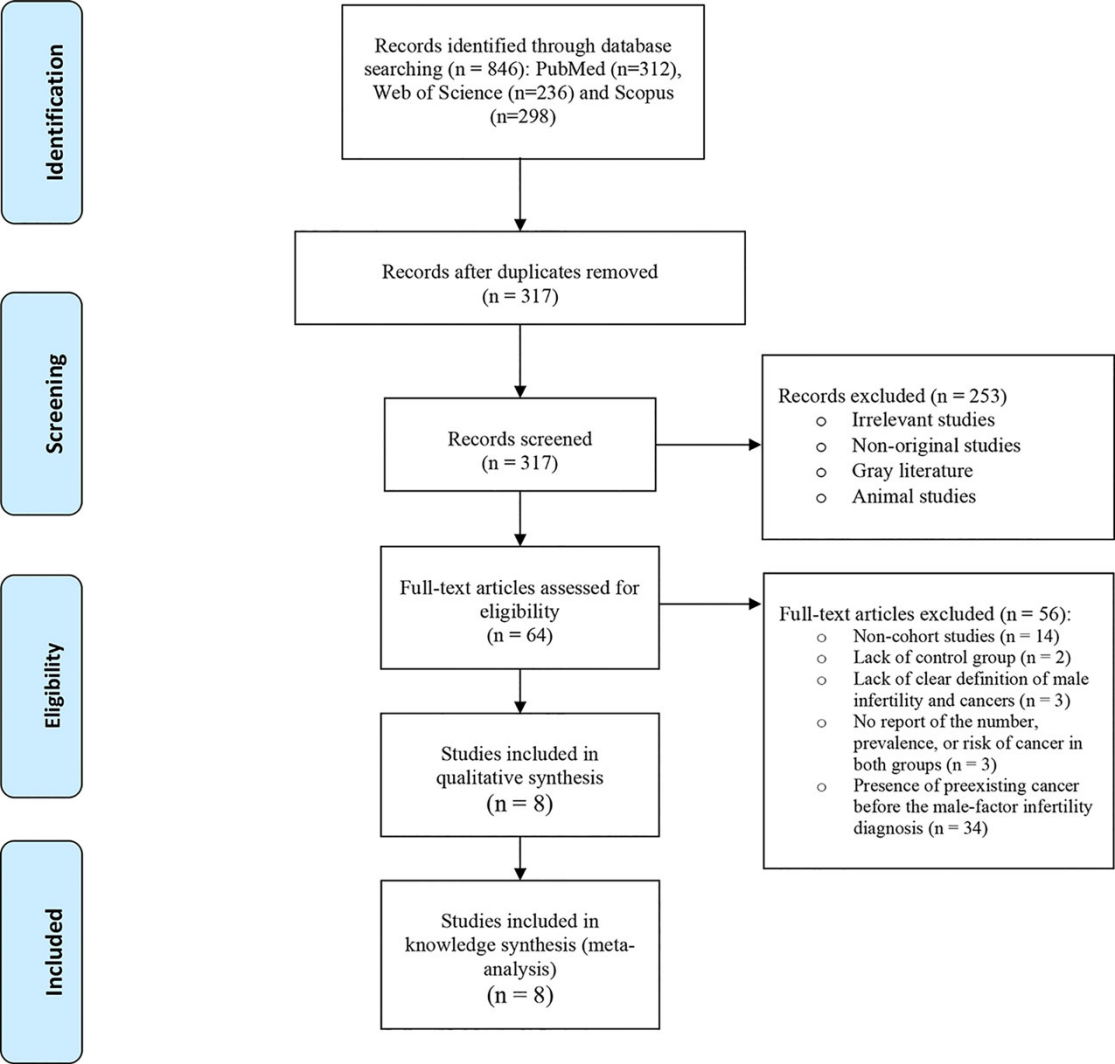
Flow diagram of the search strategy and study selection.

**Figure 1** illustrates the flow diagram of the search strategy and study selection. In the initial search, 846 studies were retrieved. Of which, 529 articles were duplicate appearing in multiple databases, which were excluded. During abstract reading, 253 studies were excluded because they were irrelevant, did not examine male infertility, or contained in-vivo examinations. The full-text of 64 remaining studies were assessed and 56 studies that did not meet the inclusion criteria were also excluded. The remaining studies (n=8) were selected for the final research analyses consisting of 168,327 men with the diagnosis of infertility and 2,252,806 men without it. With regard to their methodologies, 7 were retrospective studies (19–25) and one was prospective cohort study (11). A total of 5 studies were conducted in the USA (19–23), two in Sweden (11, 25) and one in Denmark (24). **Table 1** shows a summary of the included studies.

**Table 1:** Systemic review of the included studies in meta-analysis.

Author	Country	Study design	Male infertility Definition	Characteristics of men with infertility	Characteristics of men without infertility	Follow-up period	Control of confounder variables	Outcome of interest	Main findings
Eisenberg et al. (19)	USA	Retrospective Cohort	Men with azoospermia	N = 451 Age = 35.5 (8.3) BMI = NM	N = 1787 Age = 35.8 (6.9) BMI = NM	6.7 years	Adjusted for age and year of evaluation	All cancers	Compared to the general population, men with infertility had a higher risk of cancer. Stratifying by the azoospermia status showed that azoospermic men had an elevated risk of cancer, and those without azoospermia had a trend toward a higher rate of cancer
Eisenberg et al. (20)	USA	Retrospective Cohort	ICD-9	N = 76083 Age = 35.08 (5.89) BMI = NM	N = 760830 Age = NM BMI = NM	277703 person-years	Matched on age and follow-up time	All and individual cancers of testicular, prostate, melanoma, kidney, upper aerodigestive, stomach, colon and rectum, liver and gallbladder, pancreas, urinary bladder, breast and lung, esophagus, leukemia, Hodgkin lymphoma, non-Hodgkin lymphoma, thyroid, nervous system	Men with infertility had a higher risk of testicular cancer, non-Hodgkin lymphoma and all cancers than the counterparts men without infertility
Elenkov et al. (11)	Sweden	Prospective population-based cohort	Being childless	N = 2134 Age = 48.9 (4.1) BMI = 25.3 (3.8)	N = 9209 Age = 48.7 (3.9) BMI = 25 (3.2)	Up to 42 years	Adjusted for smoking, education and marital status, BMI, high blood pressure	Prostate cancer	Childless men had the higher risk of prostate cancer-related mortality compared to men with children. However, the prostate cancer incidence did not differ between them
Hanson et al. (21)	USA	Retrospective cohort	All men presenting for infertility that underwent semen analysis	N = 20433 Age = NM BMI = NM	N = 20433 Age = NM BMI = NM	On average for 7.3 years with a maximum of 18 years	Matched on age and birth year	All cancers, and prostate, testicular, melanoma, and other cancers	Men with semen analysis had an increased risk of testicular cancer compared to men without infertility. There were no significant differences in the cancer risk for the other common sites or the overall risk of cancer
Walsh et al. (22)	USA	Retrospective cohort	Infertile men with abnormal semen WHO-1999 criteria	N = 4549 Age = 38.1 (7.4) BMI = NM	N = 14557 Age = 36.4 (6.4) BMI = NM	Mean (SD): 11.4 (2.9) years	Matched on age	Testicular Cancer	Men with infertility had an increased risk of subsequently developing testicular cancer
Walsh et al. (23)	USA	Retrospective cohort	Infertile men with abnormal semen WHO-1999 criteria	N = 4549 Age = 38.1 (7.4) BMI = NM	N = 14557 Age = 36.4 (6.4) BMI = NM	Mean (SD): 11.4 (2.9) years	Matched on age and geography	Prostate Cancer	Men with male infertility were found to have an increased risk of subsequently developing high-grade prostate cancer
Jacobsen et al. (24)	Denmark	Retrospective cohort	All men in couples with fertility problems who had impaired semen analysis	N = 29177 Age = NM BMI = NM	N = 300000 Age = NM BMI = NM	NM	NM	All cancers, and testicular, peritoneum and other cancers	Men with infertility were more likely to develop testicular and peritoneum and other digestive organs cancer than other men
Al-Jebari et al. (25)	Sweden	Retrospective cohort	All men used assisted reproductive techniques including IVF and ICSI	N = 35500 Age = NM BMI = NM	N = 1145990 Age = NM BMI = NM	14389198 person years	Adjusted for fathers’ age at childbirth, father’s education level	Prostate cancer	Men who became fathers through assisted reproduction had a statistically significantly increased risk of prostate cancer compared with men who conceived naturally

ICD, International Statistical Classification of Diseases; 9th edition; BMI, Body Mass Index; IVF, In vitro fertilization; ICSI, Intracytoplasmic sperm injection; NM, Not mentioned.

Four studies assessed the risk of testicular cancer (20–22, 24), five evaluated prostate cancer (11, 20, 21, 23, 25), two examined melanoma (20, 21). In addition, three studies reported the other types of oncologic outcomes of kidney, upper aerodigestive, stomach, colon and rectum, liver and gallbladder, pancreas, urinary bladder, breast and lung, esophagus, leukemia, Hodgkin lymphoma, non-Hodgkin lymphoma, thyroid, nervous system, peritoneum, and rest of cancers (20, 21, 24) and one study reported the risk of all cancers in both men with and without infertility (19). The quality assessment of the included studies has been presented in **Supplementary Table 3**. All studies were classified as high quality.

### Meta-Analysis of Outcomes

**Table 2** shows the pooled OR of single and composite cancers, estimation of heterogeneity, and assessment of publication bias in both groups.

**Table 2:** Results of heterogeneity estimation and publication bias assessment, and meta-analysis for the risk of cancer among men with infertility compared to men without infertility.

Outcome		Sample size				Publication bias test			Heterogeneity(I^2^%)*	Pooled overall OR (95% CI)*
		Men with infertility		Men without infertility		Harbord test	Egger test	Begg test		
		Total population	Number of events	Total population	Number of events					
Composite all cancers		168327	1551	2252806	12164	0.365	0.188	0.697	**77.1%**	**1.43 (1.25-1.64)**
Individual cancers										
	Prostate cancer	138699	324	1951019	3875	0.348	0.392	1.000	**79.9%**	**1.48 (1.05-2.08)**
	Testicular cancer	130242	183	1095820	849	0.497	0.152	0.497	30.4%	**1.91 (1.52-2.42)**
	Melanoma cancer	96516	121	781263	540	Insufficient data	Insufficient data	0.317	0.0%	**1.31 (1.06-1.62)**

*Bold values indicate statistical significance.

**Figure 2:**
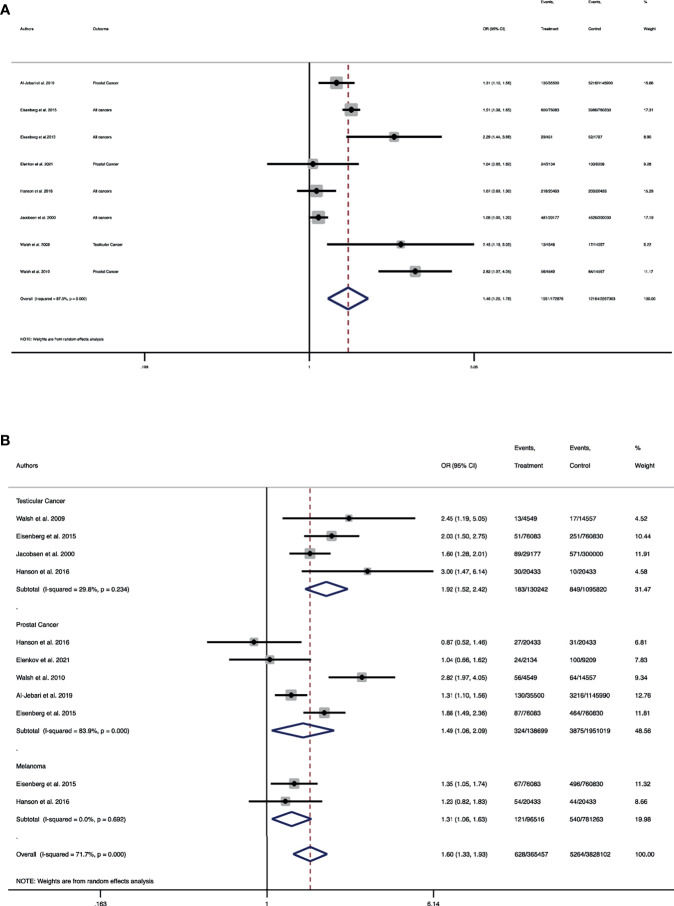
Forest plot of pooled odds ratio for **(A)** composite outcome of all cancers; **(B)** individual cancers of testicular, prostate and melanoma. **(A)** Forest plot of the pooled odds ratio for the composite outcome of all cancers. **(B)** Forest plot of pooled odds ratio for testicular cancer, prostate cancer and melanoma.

In term of composite outcome of cancers, a total of 8 studies involving 1,585,940 men with infertility and 15,862,783 men without infertility were entered into the meta-analysis. The pooled OR of cancers, regardless of type of cancer, among men with the diagnosis of infertility was 1.4 folds higher than men without it (Pooled OR = 1.46, 95% CI: 1.20-1.78) (**Figure 2A**). Meta-analysis of individual cancers was conducted and compared for prostate, testicular and melanoma between the groups. A total of 4 studies including 130,242 men with male infertility and 1,095,820 without it were entered into the meta-analysis of testicular cancer. The risk of testicular cancer in men with male infertility was 1.9 folds higher than men without infertility (Pooled OR = 1.91, 95% CI: 1.52-2.42) (**Figure 2B**).

Regarding prostate cancer, a total of 5 studies involving 138,699 men with infertility and 1,951,019 men without infertility were included in the meta-analysis. The pooled risk of prostate cancer in men with infertility was 1.4 folds higher than men without infertility (Pooled OR = 1.48, 95% CI: 1.05-2.08) (**Figure 2B**). A total of two studies including 96,516 men with infertility and 781,263 men without infertility were entered into the meta-analysis. It was demonstrated that the pooled OR of melanoma in men with infertility was 1.3 folds higher than those without infertility (Pooled OR = 1.31, 95% CI: 1.06-1.62) (**Figure 2B**).

We performed a subgroup analysis in studies that considered impaired semen analysis for the diagnosis of male infertility. The risk of composite outcome of cancers increased in men with impaired semen analysis compared to controls (Pooled OR = 1.42, 95% CI: 1.18-1.71) (**Supplementary Figure 2**).

### Heterogeneity and Sensitivity Analysis

**Figure 3:**
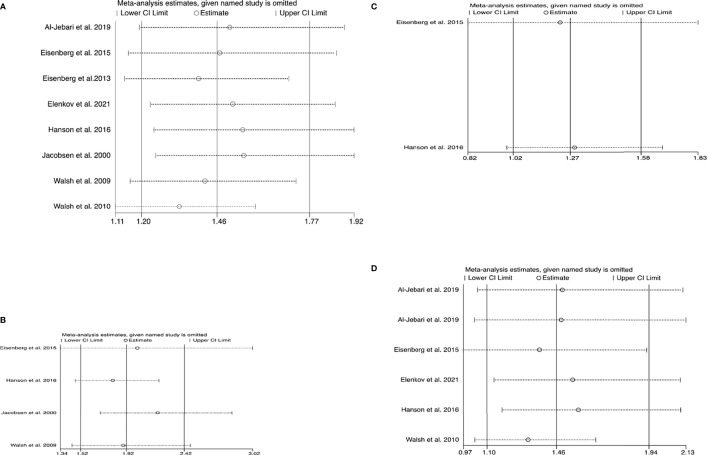
Plots of sensitivity analysis results **(A)** all cancers **(B)** testicular cancer **(C)** melanoma **(D)** prostate cancer. These graphs show the influence of each individual study on the overall meta-analysis summary estimate. Accordingly, the results of an influence analysis in which the meta-analysis is re-estimated omitting each study in turn has been shown. They provide the visual results, naming the omitted study on the left margin and omitted meta-analytic summary statistics as horizontal confidence intervals on the right side. The full, combined results have been shown as the solid vertical lines. For interpretation, an individual study is suspected of having an excessive influence if the point estimate of its omitted analysis lies outside the confidence interval of the combined analysis. **(A)** Sensitivity analysis plot for the composite outcome of cancers. **(B)** Sensitivity analysis plot for testicular cancer. **(C)** Sensitivity analysis plot for melanoma. **(D)** Sensitivity analysis plot for prostate cancer.

No statistically significant heterogeneity was found in the studies with regard to the assessment of testicular cancer and melanoma (all I^2^ < 50%, P > 0.05), whereas heterogeneity was observed among the studies with regard to the analysis of the composite outcome of all cancers and prostate cancer (all I^2^ > 50%, P < 0.01) (**Table 2**). However, sensitivity analysis showed that no single study essentially changed the pooled OR of all outcomes (**Figures 3A–D**).

### Publication Bias and Risk of Bias

**Figure 4:**
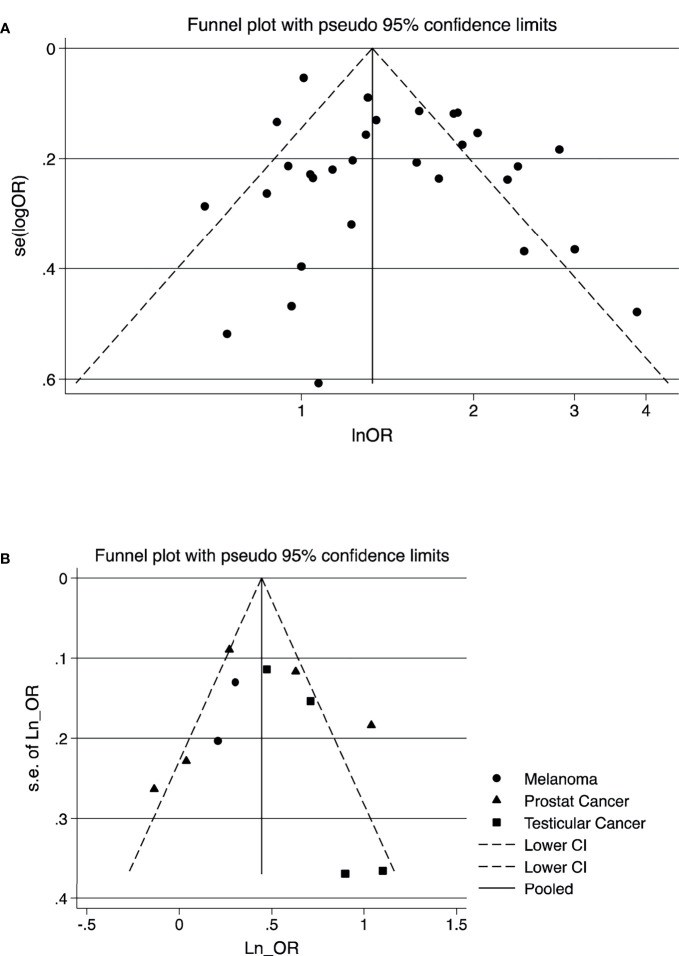
Funnel plot for the visual assessment of publication bias of the outcome of studies; **(A)** composite outcome of all cancers **(B)** testicular cancer, melanoma, and prostate cancer. **(A)** Composite outcome of cancers. **(B)** Testicular cancer, melanoma, and prostate cancer.

According to the results of publication bias tests, no substantial publication bias for meta-analysis was observed (Table 2), which also was confirmed by the symmetric funnel plot (**Figures 4A, B**). Additionally, the included studies mostly were judged as having a low risk of bias for the evaluated domains. All studies had low or moderate of bias for all domains of selection of exposed and non-exposed cohorts, assessment of exposure, presence of the outcome of interest at the beginning of the study, outcome assessment, assessment of the presence or absence of prognostic factors, and adequacy of follow up of cohorts. However, approximately, 10% had a serious risk of bias in controlling prognostic variables (**Supplementary Figures 1A, B**).

## Discussion

Despite the insufficient number of studies for the precise comparison of the different types of cancers in males with the diagnosis of infertility, the present systematic review and meta-analysis based on available evidence revealed that the risk of cancer regardless of the type of cancer and also the risk of individual cancers of testis, melanoma, and prostate increased in men with the diagnosis of infertility.

Male infertility is a heterogeneous, complex, and mostly multifactorial problem. The exact etiology of male infertility is unknown, with half of cases classified as idiopathic or unexplained (26). Similarly, the association between male infertility and the occurrence of cancer has been poorly understood. However, it has been hypothesized that a complex interaction between genetics and epigenetics, developmental, and lifestyle or environmental factors can put men with infertility at the risk of developing cancer in the future (9, 11). In this respect, at least 1500 genes are known that contribute to spermatogenesis and any defect in these genes may also potentially lead to the development of infertility, male genitourinary, and cancer in other organs (9, 27). Nagirnaja et al. stated that disturbances in cell survival, cell fate, and genome maintenance might be a shared biological process in both male infertility and cancer. Moreover, there were at least 25 tumor-suppressor genes or oncogenes with a potentially pleomorphic effect that contributed to both male infertility and the development of a malignancy (28). Although clear evidence is available in mice, more studies are needed to explain related associations in humans. In addition, the environmental toxin exposure, and also prenatal exposure to commonly used chemicals, e.g. phthalates, may increase the risk of both infertility and neoplasm in men (29).

Additionally, it has been reported that the fear of infertility and its consequences may influence help-seeking behaviors in men, which may trigger diseases in later life (30). The experience of infertility may negatively impact on personal health behaviors (31) and in some contexts, it may hinder taking further actions to prevent health-related problems or seek appropriate treatments (29). It has been hypothesized that unhealthy behaviors may predispose infertile patients to cancer. However, this hypothesis could not be tested in our study and should be considered in future studies. Nevertheless, early educational programs on men’s health as inexpensive prevention strategies in the community should be used to inform them of related health issues and how to seek appropriate treatments (32).

Prior studies have focused on the relationship between the infertility status and male-specific malignancies. Mao et al. conducted a systemic review and meta-analysis on the association between the fatherhood status and the risk of prostate cancer, and found that the risk of prostate cancer were lower among childless men (OR = 0.91, 95% CI: 0.87–0.96) (13). However, the results of this study should be interpreted with caution because heterogeneity in the definition of infertility and presence of selection bias might have distorted pooled estimates. In addition, half of the included studies had the case-control design, which could be affected by selection bias and recall bias. Another meta-analysis by Del Giudice et al. (12) examined the correlation between impaired male fertility and the risk of developing testicular and prostate cancers in cohort studies. They reported that male infertility was significantly associated with the subsequent risk of testicular cancer (RR = 1.68, 95% CI: 1.17-2.4) and prostate cancer (RR = 2.03, 95% CI: 1.66-2.48) (12). Although narrow inclusion criteria for this study led to the adoption of a small number of studies to this meta-analysis, its findings were in agreement with the finding of our meta-analysis. The findings of our meta-analysis comprehensively adds new knowledge to the body of international literature and also helps with the provision of an updated evidence on this important topic.

Our study have some limitations that should be considered in the interpretation of data. In our review, a considerable heterogeneity between the studies that assessed the risk of prostate cancer was observed. Different criteria for the screening and recruitment of infertile men in the included studies, lack of uniform data regarding risk stratification such as the grade and stage of cancers, and absence of risk factors such as race/ethnicity, socio-economic and demographics may have led to the heterogeneity. Therefore, definitive conclusions should be taken cautiously. Also, the included studies had large sample sizes in case and control groups, but the number of studies assessing the occurrence of cancer in men with infertility was limited and hindered us to run various subgroup analyses for the different types of cancers, stages of cancer, and the degree of male infertility impairment. Moreover, various confounders for the development of cancer such as lifestyle factors (33) could not be accounted for in this meta-analysis due to the lack of sufficient data about them in the included studies. Information about cancer in the included studies was extracted from databases that might have caused bias in our results. There is the possibility of duplicate populations between the included studies conducted in the USA, since some of them used databases that covered the same population. Gray literature could be the important source of available knowledge, which were excluded from our literature search due to the lack of peer review process and methodological descriptions and details, which made it difficult to evaluate their quality.

## Conclusion

This systematic review and meta-analysis demonstrated an increased risk of composite outcome of cancers as well as melanoma, testicular and prostate cancers in men with the diagnosis of male infertility. Well-designed long-term prospective studies, considering all known cancers and their accompanying risk factors should be conducted to support our findings.

## Data Availability Statement

The original contributions presented in the study are included in the article/**Supplementary Material**. Further inquiries can be directed to the corresponding author.

## Author Contributions

SB-G conceptualized the study and was involved in study design, search on databases, study selection, data extraction, drafting the manuscript, and revising it critically for important intellectual content. MV was involved in study design, study selection, data extraction, manuscript drafting, editing, and revising it critically for important intellectual content. RB-Y contributed to quality assessment, data analysis, interpreting data and drafting the manuscript. MP contributed to quality assessment, data analysis, and interpreting data. All authors contributed to the article and approved the submitted version.

## Funding

Nord University, Bodø, Norway covered the article processing charge.

## Conflict of Interest

The authors declare that the research was conducted in the absence of any commercial or financial relationships that could be construed as a potential conflict of interest.

## Publisher’s Note

All claims expressed in this article are solely those of the authors and do not necessarily represent those of their affiliated organizations, or those of the publisher, the editors and the reviewers. Any product that may be evaluated in this article, or claim that may be made by its manufacturer, is not guaranteed or endorsed by the publisher.

## References

[1] Zegers-Hochschild F Adamson GD de Mouzon J Ishihara O Mansour R Nygren K . International Committee for Monitoring Assisted Reproductive Technology (ICMART) and the World Health Organization (WHO) Revised Glossary of ART Terminology, 2009. Fertil Steril (2009) 92(5):1520–4. doi: 10.1016/j.fertnstert.2009.09.009 19828144

[2] Kazemijaliseh H Ramezani Tehrani F Behboudi-Gandevani S Hosseinpanah F Khalili D Azizi F . The Prevalence and Causes of Primary Infertility in Iran: A Population-Based Study. Glob J Health Sci (2015) 7(6):226–32. doi: 10.5539/gjhs.v7n6p226 PMC4803880 26153187

[3] Boivin J Bunting L Collins JA Nygren KG . International Estimates of Infertility Prevalence and Treatment-Seeking: Potential Need and Demand for Infertility Medical Care. Hum Reprod (2007) 22(6):1506–12. doi: 10.1093/humrep/dem046 17376819

[4] Eisenberg ML Li S Behr B Cullen MR Galusha D Lamb DJ . Semen Quality, Infertility and Mortality in the USA. Hum Reprod (Oxford England) (2014) 29(7):1567–74. doi: 10.1093/humrep/deu106 PMC4059337 24838701

[5] Glazer CH Bonde JP Eisenberg ML Giwercman A Hærvig KK Rimborg S . Male Infertility and Risk of Nonmalignant Chronic Diseases: A Systematic Review of the Epidemiological Evidence. Semin Reprod Med (2017) 35(3):282–90. doi: 10.1055/s-0037-1603568 28658712

[6] Rogers MJ Walsh TJ . Male Infertility and Risk of Cancer. Semin Reprod Med (2017) 35(3):298–303. doi: 10.1055/s-0037-1603583 28658714

[7] Martins AD Majzoub A Agawal A . Metabolic Syndrome and Male Fertility. World J Mens Health (2019) 37(2):113–27. doi: 10.5534/wjmh.180055 PMC6479081 30350486

[8] Bungum AB Glazer CH Bonde JP Nilsson PM Giwercman A Søgaard Tøttenborg S . Risk of Metabolic Disorders in Childless Men: A Population-Based Cohort Study. BMJ Open (2018) 8(8):e020293–e. doi: 10.1136/bmjopen-2017-020293 PMC6104745 30121591

[9] Kasman AM Del Giudice F Eisenberg ML . New Insights to Guide Patient Care: The Bidirectional Relationship Between Male Infertility and Male Health. Fertil Steril (2020) 113(3):469–77. doi: 10.1016/j.fertnstert.2020.01.002 32089256

[10] Behboudi-Gandevani S Bidhendi Yarandi R Rostami Dovom M Azizi F Ramezani Tehrani F . The Association Between Male Infertility and Cardiometabolic Disturbances: A Population-Based Study. Int J Endocrinol Metab (2021) 19(2):e107418. doi: 10.5812/ijem.107418 34149845 PMC8198602

[11] Elenkov A Giwercman A Zhang H Nilsson PM Giwercman YL . Increased Risk for Prostate Cancer Related Mortality Among Childless Men in a Population-Based Cohort Followed for Up to 40 Years. Scand J Urol (2021), 55(2):125–8. doi: 10.1080/21681805.2021.1889027 33615988

[12] Del Giudice F Kasman AM De Berardinis E Busetto GM Belladelli F Eisenberg ML . Association Between Male Infertility and Male-Specific Malignancies: Systematic Review and Meta-Analysis of Population-Based Retrospective Cohort Studies. Fertil Steril (2020) 114(5):984–96. doi: 10.1016/j.fertnstert.2020.04.042 32709378

[13] Mao Y Xu X Zheng X Xie L . Reduced Risk of Prostate Cancer in Childless Men as Compared to Fathers: A Systematic Review and Meta-Analysis. Sci Rep (2016) 6:19210. doi: 10.1038/srep19210 26752096 PMC4707492

[14] Page MJ McKenzie JE Bossuyt PM Boutron I Hoffmann TC Mulrow CD . Updating Guidance for Reporting Systematic Reviews: Development of the PRISMA 2020 Statement. J Clin Epidemiol (2021) 134:103–12. doi: 10.1016/j.jclinepi.2021.02.003 33577987

[15] Barratt CLR Björndahl L De Jonge CJ Lamb DJ Osorio Martini F McLachlan R . The Diagnosis of Male Infertility: An Analysis of the Evidence to Support the Development of Global WHO Guidance-Challenges and Future Research Opportunities. Hum Reprod Update (2017) 23(6):660–80. doi: 10.1093/humupd/dmx021 PMC5850791 28981651

[16] Wells GA Shea B O’Connell D Peterson J Welch V Losos M . The Newcastle-Ottawa Scale (NOS) for Assessing the Quality of Nonrandomized Studies in Meta-Analyses. Ottawa, ON, Canada: Ottawa Hospital Research Institute. Available at: http://www.ohri.ca/programs/clinical_epidemiology/oxford.asp (Accessed on 25 Feb 2021).

[17] Sterne JA Hernán MA Reeves BC Savović J Berkman ND Viswanathan M . ROBINS-I: A Tool for Assessing Risk of Bias in Non-Randomised Studies of Interventions. BMJ (2016) 355:i4919. doi: 10.1136/bmj.i4919 27733354 PMC5062054

[18] (2011).

[19] Eisenberg ML Betts P Herder D Lamb DJ Lipshultz LI . Increased Risk of Cancer Among Azoospermic Men. Fertil Steril (2013) 100(3):681–5. doi: 10.1016/j.fertnstert.2013.05.022 PMC3759541 23790640

[20] Eisenberg ML Li S Brooks JD Cullen MR Baker LC . Increased Risk of Cancer in Infertile Men: Analysis of U.S. Claims Data. J Urol (2015) 193(5):1596–601. doi: 10.1016/j.juro.2014.11.080 25463997

[21] Hanson HA Anderson RE Aston KI Carrell DT Smith KR Hotaling JM . Subfertility Increases Risk of Testicular Cancer: Evidence From Population-Based Semen Samples. Fertil Steril (2016) 105(2):322–8.e1. doi: 10.1016/j.fertnstert.2015.10.027 PMC4744156 26604070

[22] Walsh TJ Croughan MS Schembri M Chan JM Turek PJ . Increased Risk of Testicular Germ Cell Cancer Among Infertile Men. Arch Intern Med (2009) 169(4):351–6. doi: 10.1001/archinternmed.2008.562 PMC2881689 19237718

[23] Walsh TJ Schembri M Turek PJ Chan JM Carroll PR Smith JF . Increased Risk of High-Grade Prostate Cancer Among Infertile Men. Cancer (2010) 116(9):2140–7. doi: 10.1002/cncr.25075 PMC2893877 20309846

[24] Jacobsen R Bostofte E Engholm G Hansen J Olsen JH Skakkebaek NE . Risk of Testicular Cancer in Men With Abnormal Semen Characteristics: Cohort Study. BMJ (2000) 321(7264):789–92. doi: 10.1136/bmj.321.7264.789 PMC27489 11009515

[25] Al-Jebari Y Elenkov A Wirestrand E Schütz I Giwercman A Lundberg Giwercman Y . Risk of Prostate Cancer for Men Fathering Through Assisted Reproduction: Nationwide Population Based Register Study. BMJ (2019) 366:l5214. doi: 10.1136/bmj.l5214 31554611 PMC6759809

[26] Hanson BM Eisenberg ML Hotaling JM . Male Infertility: A Biomarker of Individual and Familial Cancer Risk. Fertil Steril (2018) 109(1):6–19. doi: 10.1016/j.fertnstert.2017.11.005 29307404

[27] Aston KI Conrad DF . A Review of Genome-Wide Approaches to Study the Genetic Basis for Spermatogenic Defects. Methods Mol Biol (2013) 927:397–410. doi: 10.1007/978-1-62703-038-0_34 22992931

[28] Nagirnaja L Aston KI Conrad DF . Genetic Intersection of Male Infertility and Cancer. Fertil Steril (2018) 109(1):20–6. doi: 10.1016/j.fertnstert.2017.10.028 PMC5761685 29307395

[29] Zarif Golbar Yazdi H Aghamohammadian Sharbaf H Kareshki H Amirian M . Psychosocial Consequences of Female Infertility in Iran: A Meta-Analysis. Front Psychiatry (2020) 11:518961. doi: 10.3389/fpsyt.2020.518961 PMC7674496 33250787

[30] Boivin J Carrier J Zulu JM Edwards D . A Rapid Scoping Review of Fear of Infertility in Africa. Reprod Health (2020) 17(1):142 doi: 10.1186/s12978-020-00973-0 32928239 PMC7488744

[31] Datta J Palmer MJ Tanton C Gibson LJ Jones KG Macdowall W . Prevalence of Infertility and Help Seeking Among 15 000 Women and Men. Hum Reprod (Oxford England) (2016) 31(9):2108–18. doi: 10.1093/humrep/dew123 PMC4991655 27365525

[32] Jeihooni AK Kashfi SM Hatami M Avand A Bazrafshan MR . The Effect of Educational Program Based on PRECEDE Model in Promoting Prostate Cancer Screening in a Sample of Iranian Men. J Cancer Educ (2019) 34(1):161–72. doi: 10.1007/s13187-017-1282-8 28913671

[33] Katzke VA Kaaks R Kühn T . Lifestyle and Cancer Risk. Cancer J (2015) 21(2):104–10. doi: 10.1097/PPO.0000000000000101 25815850

