# Supplementary material for: A Systematic Review and Meta-Analysis of Male Infertility and the Subsequent Risk of Cancer
Source: Front Oncol. 2021 Oct 14;11:696702. doi: 10.3389/fonc.2021.696702 (PMC8551623; doi:10.3389/fonc.2021.696702)
Supplement: Supplementary file 1 [file DataSheet_1.docx]

**Supplementary file**

Supplementary table 1. The protocol for the systematic review of male infertility and the subsequent risk of cancer

| **Review title** | Male infertility and risk of developing cancer: A systematic review and meta-analysis |
| --- | --- |
| **PICO (population, intervention/Index, control, and outcomes) statement** | The risk of developing overall and individual cancer among men with the diagnosis of male factor infertility compared to those who do not suffer from male-factor infertility.  P: men with the diagnosis of male factor infertility; I: risk of developing cancer; C: those men who do not suffer from male-factor infertility; O: overall and individual cancer. |
| **Anticipated or actual start date** | 01.2.2021 |
| **Anticipated completion date** | 01.6.2021 |
| **Review team details and rolls** | - Associate Professor, Samira Behboudi-Gandevani, Faculty of Health Sciences, Nord University, Bodø, Norway, Corresponding author. She conceptualizes the study and is involved in study design, search in databases, study selection, data extraction, drafting the manuscript, and revising it critically for important intellectual content. - Assistant Professor, Razieh Bidhendi-Yarandi, University of Social Welfare and Rehabilitation Sciences, Tehran, Iran, She contributes to quality assessment, data analysis, interpreting data and drafting the manuscript. - Assistant Professor Mohammad Hossein Panahi, School of Public Health and Safety, Shahid Beheshti University of Medical Sciences, Tehran, Iran. He contributes to quality assessment, data analysis, interpreting data and drafting the manuscript. - Professor Mojtaba Vaismoradi, Faculty of Health Sciences, Nord University, Bodø, Norway. He is involved in study design, search in databases, study selection, data extraction, manuscript drafting, editing, and revising it critically for important intellectual content. |
| **Funding sources/sponsors** | None. This Study is performed in the framework of the research time of faculty members. |
| **Conflicts of interest** | None. |
| **Aim of study** | The primary objective is to investigate the risk of developing composite outcome of all cancers, regardless of type of cancer among men with infertility diagnosis compared to fertile counterparts.  The secondary objective is to examine the pooled risk of developing individual specific cancer among these population. |
| **Identification of the research question** | - What is the pooled risk of developing composite outcome of all cancers, regardless of type of cancer among men with infertility diagnosis? - What is the pooled risk of developing individual specific cancers among men with the diagnosis of infertility? |
| **Searches** | The following electronic bibliographic databases are searched: PubMed (including Medline), Scopus, and Web of Science to retrieve relevant studies.  A manual search in the references list of selected studies and other relevant reviews will be carried out to maximize the identification of eligible studies.  A librarian at Nord University assists with the search process. |
| **Language** | The search strategy is restricted to English language with no time limitations. |
| **Keywords** | Keywords are as follows: (male infertility OR male sterility OR male sub-fertility OR azoospermia OR oligospermia OR semen quality OR fertility impairment) AND (cancer OR neoplasms OR neoplasia OR tumors OR carcino* OR onco* OR benign OR hyperplasia OR malignancies OR malignancy OR carcinoma). |
| **Eligibility criteria** | Inclusion Criteria:  (i) all types of observational cohort studies including prospective and retrospective  (ii) registered-based data  (iii) all studies should have the control group as subjects without infertility  (iv) report of a clear definition of male infertility and cancers, number, prevalence or risk of cancer in cases and controls.  Exclusion Criteria:  (i) Non-original studies including reviews, commentaries, editorials, letters, meeting abstracts, case reports, and conference proceedings  (ii) those do not provide accurate and clear data on research variables  (iii) the presence of pre-existing cancer before the male-factor infertility diagnosis  (iv) studies without indicating any difference between women and men infertility  (v) Gray literature including governmental or organizational reports, conference proceedings, dissertations, theses, unpublished data, presentations, etc |
| **Condition or domain being studied** | Risk of developing cancer in men with male infertility diagnosis. |
| **Primary outcome(s)** | Primary outcome of interest is the pooled risk of developing composite outcome of all cancers, regardless of type of cancer among men with infertility diagnosis. |
| **Secondary outcome(s)** | Secondary outcome is the pooled risk of developing individual specific cancers among men with the diagnosis of infertility. |
| **Search strategy** | Search is performed on the mentioned databases for retrieving observational studies published up to 31. Feb. 2021, which investigates the risk of the development of cancer in males with the diagnosis of infertility. Further, a manual search in the references list of selected studies and other relevant reviews is performed to maximize the search coverage. |
| **Data extraction (selection and coding)** | The following data are extracted: the first author’s name; journal title; publication year; country; study design; sample size; population characteristics including age and body mass index; infertility definition; cancer definition, follow-up period, quality assessment and outcome measurements including the number and prevalence of cancer.  The accuracy of gathered data before the meta-analysis is assessed through double checking the data extraction process to ensure of no bias in the data extraction and data entry. |
| **Risk of bias and quality assessment** | Two review authors independently assess the quality and risk of bias in included studies by considering the following characteristics:  Quality of the included studies is appraised using the modification of the Newcastle–Ottawa Quality Assessment Scale for Non-Randomized Studies with regard to selection, comparability and outcomes. Studies with scores above 6 are considered high quality, 4–6 moderate quality, and less than 4 low quality.  The (ROBINS) tool in non-randomized studies of interventions and observational studies is used to assess the risk of bias. Seven domains of (i) selection of exposed and non-exposed cohort, (ii) assessment of exposure, (iii) presence of outcome of interest at the start of the study, (iv) control of prognostic variables, (v) assessment of the presence or absence of prognostic factors, (vi) assessment of outcome, (vii) adequacy of follow-up are used during appraisal. The authors classify their judgment about the quality of each study into critical, moderate risk, and low risk of bias. |
| **Strategy for data synthesis** | A narrative synthesis of the findings is performed from the included studies, structured around the type of studies, target population characteristics, type of outcome and its content.  Meta-analysis is conducted to evaluate the pooled OR (95% CI) of the outcomes of interest using the DerSimonian and Laird and inverse variance methods.  Heterogeneity and publication bias analyses are also evaluated.  Sensitivity analysis is run to investigate the influence of each individual study on the overall meta-analysis summary estimate.  Subgroup analysis is performed based on available data about individual cancer.  R and STATA software is used for meta-analyses. |
| **Equator guidelines** | The Preferred Reporting Items Systematic Reviews and Meta-analysis (PRISMA) |

Supplementary table 2. Detailed Search Syntax for electronic databases.

| PubMed (including Medline) | Search: #1 AND #2 Filters: Journal Article, English  Search #1: cancer[Title/Abstract] OR neoplasms[Title/Abstract] OR neoplasia[Title/Abstract] OR tumors[Title/Abstract] OR carcino[Title/Abstract] OR onco[Title/Abstract] OR benign[Title/Abstract] OR hyperplasia[Title/Abstract] OR malignancies[Title/Abstract] OR malignancy[Title/Abstract] OR carcinoma[Title/Abstract] Filters: Journal Article, English  Search #2: male infertility[Title/Abstract] OR male sterility[Title/Abstract] OR male sub-fertility[Title/Abstract] OR Azoospermia[Title/Abstract] OR Oligospermia[Title/Abstract] Filters: Journal Article, English |
| --- | --- |
| Web of Science | # 3  (#1 AND #2) AND LANGUAGE: (English) AND DOCUMENT TYPES: (Article)  Indexes=SCI-EXPANDED, SSCI, A&HCI, ESCI Timespan=All years    # 2  (TS=(cancer OR neoplasms OR neoplasia OR tumors OR carcino OR onco OR benign OR hyperplasia OR malignancies OR malignancy OR carcinoma) ) AND LANGUAGE: (English) AND DOCUMENT TYPES: (Article)  Indexes=SCI-EXPANDED, SSCI, A&HCI, ESCI Timespan=All years    # 1  (TS=(male infertility OR male sterility OR male sub-fertility OR Azoospermia OR Oligospermia) ) AND LANGUAGE: (English) AND DOCUMENT TYPES: (Article)  Indexes=SCI-EXPANDED, SSCI, A&HCI, ESCI Timespan=All years |
| Scopus | TITLE-ABS-KEY ( ( male AND infertility OR male AND sterility OR male AND sub-fertility OR azoospermia OR oligospermia ) AND ( cancer OR neoplasms OR neoplasia OR tumors OR carcino OR onco OR benign OR hyperplasia OR malignancies OR malignancy OR carcinoma ) ) AND ( LIMIT-TO ( DOCTYPE , "ar" ) ) AND ( LIMIT-TO ( LANGUAGE , "English" ) ) |

Supplementary table 3. Quality assessment of included studies using the Newcastle–Ottawa Quality Assessment Scale for cohort studies.

|  | SELECTION | | | | COMPARABILITY | OUTCOME | | | Total scores |
| --- | --- | --- | --- | --- | --- | --- | --- | --- | --- |
| Author | Representativeness of the exposed cohort | Selection of the non-exposed cohort | Ascertainment of exposure | Demonstration that the outcome of interest was not present at the start of study | A: Study controls for age and/or Sex  B: Study controls for other confounders | A: Independent blind assessment  B: Record linkage | follow-up long enough for outcomes | Adequacy of follow up of cohorts |  |
| Al-Jebari et al. 2019 (26) | * | * | * | * | ** | * | * | * | 9 |
| Eisenberg et al. 2015 (21) | * | * | * | * | ** | * | * | * | 9 |
| Eisenberg et al. 2013 (20) | * | * | * | * | * | * | * | * | 8 |
| Elenkov et al. 2021 (27) | * | * | * | * | ** | * | * | * | 9 |
| Hanson et al. 2016 (22) | * | * | * | * | * | * | * | * | 8 |
| Jacobsen et al. 2000 (25) | * | * | * | * | - | * | * | * | 7 |
| Walsh et al. 2010 (24) | * | * | * | * | * | * | * | * | 8 |
| Walsh et al. 2009 (23) | * | * | * | * | * | * | * | * | 8 |

**Supplementary Figure 1.** Risk of bias in cohort studies.

A:

| First author, date | Bias in the selection of exposed and non-exposed cohorts | Bias in the assessment of exposure | Bias in the presence of outcome of interest at start of study | Bias in the control of prognostic variables (with matching or adjusting) | Bias in the assessment of the presence or absence of prognostic factors | Bias in the assessment of outcome | Bias in adequacy about follow up of cohorts |  |
| --- | --- | --- | --- | --- | --- | --- | --- | --- |
| Al-Jebari et al. 2019 (26) |  |  |  |  |  |  |  |  |
| Eisenberg et al. 2015 (21) |  |  |  |  |  |  |  |  |
| Eisenberg et al. 2013 (20) |  |  |  |  |  |  |  |  |
| Elenkov et al. 2021 (27) |  |  |  |  |  |  |  |  |
| Hanson et al. 2016 (22) |  |  |  |  |  |  |  |  |
| Jacobsen et al. 2000 (25) |  |  |  |  |  |  |  |  |
| Walsh et al. 2010 (24) |  |  |  |  |  |  |  |  |
| Walsh et al. 2009 (23) |  |  |  |  |  |  |  |  |
| Low risk of bias (the study is comparable to a well-performed study)  Moderate risk of bias (the study appears to provide sound evidence but cannot be considered comparable to a well-performed study)  Serious risk of bias (the study has some important problems)  Critical risk of bias (the study is too problematic to provide any useful evidence)  No information on which to base a judgement about risk of bias. | | | | | | | | |

**B:**

Supplementary figure 2. Forest plot of pooled odds ratio for the composite outcomes of all cancers.
